# Global, regional, and national burden and quality of care index (QCI) of oral disorders: a systematic analysis of the global burden of disease study 1990–2017

**DOI:** 10.1186/s12903-023-03808-z

**Published:** 2024-01-20

**Authors:** Shervan Shoaee, Erfan Ghasemi, Ahmad Sofi-Mahmudi, Erfan Shamsoddin, Marcos Roberto Tovani-Palone, Shahin Roshani, Mohammad-Hossein Heydari, Moein Yoosefi, Masoud Masinaei, Sina Azadnaejafabadi, Esmaeil Mohammadi, Negar Rezaei, Bagher Larijani, Hossein Fakhrzadeh, Farshad Farzadfar

**Affiliations:** 1 Non-Communicable Diseases Research Center, Endocrinology and Metabolism Population Sciences Institute, Tehran University of Medical Sciences, Tehran, Iran; 2 Elderly Health Research Center, Endocrinology and Metabolism Population Sciences Institute, Tehran University of Medical Sciences, Tehran, Iran; 3 Endocrinology and Metabolism Research Center, Endocrinology and Metabolism Clinical Sciences Institute, Tehran University of Medical Sciences, Tehran, Iran; 4 Cochrane Iran Associate Centre, National Institute for Medical Research Development (NIMAD), Tehran, Iran; 5 Ribeirão Preto Medical School, University of São Paulo, São Paulo, 14049-900 Brazil; 6 The Netherlands Cancer Institute (NKI), Amsterdam, Netherlands; 7 School of Dentistry, Shahid Beheshti University of Medical Sciences, Tehran, Iran; 8 Department of Epidemiology and Biostatistics, Tehran University of Medical Sciences, Tehran, Iran

**Keywords:** Oral disorders, Tooth caries, Periodontitis, Edentulism, Burden of disease, Quality of care

## Abstract

**Background:**

Oral disorders are still a major global public health challenge, considering their perpetuating and chronic nature. Currently, there is no direct index to measure the quality of care on a population scale. Hence, we aim to propose a new index to measure the quality of care for oral disorders worldwide.

**Methods:**

We generated our database using the data from the Global Burden of Disease (GBD) study 2017. Among different variables such as prevalence, incidence, years lived with disability, and disability-adjusted life years, we utilised principal component analysis (PCA) to determine the component that bears the greatest proportion of information to generate the novel quality of care index (QCI) for oral disorders.

**Results:**

Global QCI for oral disorders gradually increased from 1990 to 2017 (from 70.5 to 74.6). No significant gender disparity was observed during this period, and the gender disparity ratio (GDR) was considered optimal in 1990 and 2017. Between 1990 and 2017, the age-standardised QCI for all oral disorders increased in all the SDI regions. The highest QCI for all oral disorders in 2017 belonged to high-middle SDI countries (=80.24), and the lowest YLDs rate was seen in the low SDI quintile. In 1990, the quality of care in European, Central Asian, and Central and South American countries was in the lowest quintiles, whereas the North American, East Asian, Middle Eastern, and some African countries had the highest quality of dental care. Maynmar (=100), Uganda (=92.5), Taiwan (=92.0), China (=92.5), and the United States (=89.2) were the five countries with the highest age-standardised QCI. Nicaragua (=41.3), Belgium (=40.2), Venezuela (=38.4), Sierra Leone (=30.5), and the Gambia (=30.3) were the five countries with the least age-standardised QCI values.

**Conclusion:**

The quality of care for all oral disorders showed an increasing trend on a global scale from 1990 to 2017. However, the QCI distribution was not homogenous among various regions. To prevent the exacerbation of imminent disparities in this regard, better attention to total tooth loss in high-income countries and prioritising primary healthcare provision in low-income countries are recommended for oral disorders.

**Supplementary Information:**

The online version contains supplementary material available at 10.1186/s12903-023-03808-z.

## Introduction

Oral disorders, despite being largely preventable, are still a significant global public health challenge. Dental caries is the most prevalent health condition around the globe [1, 2]. The latest Global Burden of Disease (GBD) Study estimates that around 3.5 billion people suffer from oral disorders worldwide. This number has been growing in the past few decades [3]. According to the latest estimations, total direct and indirect costs due to dental diseases were estimated to be $544.41 billion, making it the sixth most costly health condition globally in 2015 [4]. Although oral disorders are highly prevalent due to their nonfatal characteristics and mostly appearing as chronic conditions [2, 5–7], health systems often neglect them. This has mirrored in most health systems as not fully covering oral health care costs, thereby the major part of oral care being provided by the private sector [2, 8]. All these factors have been significant contributors to exacerbating the rising burden of oral diseases.

As a partially controversial concept, quantifying the quality of care has been of interest for quite a long time [9, 10]. With the presence of several approaches to tackle this issue, still, no consensus has come up concerning measuring the quality of health care. Some efforts have previously been exerted to define a framework to measure this quality. The dental quality outcomes framework in the UK in 2003 was a start to the process of incentivising quality with a primary focus on equity in access and overall health care experience [11]. These measurements’ trajectory has also shifted towards a public health standpoint using population-scale estimations [12].

Having an index to compare various regions regarding their quality of oral health care can immensely help optimise future policies and approaches, as it can represent the quality of care based on available epidemiological indices. Given that the GBD Study 2017 provides comprehensive data for 354 diseases in 195 countries worldwide and only assesses the burden of oral diseases without further diving into quality assessments [13], we aimed to measure the quality of care for oral disorders using an index we developed [3]. The oral disorders of interest in our study are dental caries (deciduous or permanent), periodontitis, edentulism or severe tooth loss, and other oral disorders based on GBD classification of oral disorders.

## Methods

Commencing the design of a multivariate index as a proxy of quality of care, we needed to estimate the following parameters: incidence, prevalence, death, years of life lost (YLLs), years lived with disability (YLDs), and disability-adjusted life years (DALYs) by cause, age, sex, year, and location. We did so by analysing all the available data from the GBD 2017 study (publicly available at: https://gbd2017.healthdata.org/gbd-search), which provides a standardised approach. More in-depth inspection of GBD 2017 methodology, including the inputs, analyses, and outputs, can be done elsewhere [13].

Unlike most oral health and dentistry studies, the reported data do not include variables such as decayed, missing, and filled teeth separately; instead, there are three variables comprising incidence, prevalence, and YLDs; categorised by age, sex, year, and location. GBD data on oral diseases (code K00-K01.1, K03-K04.99, K07-K08, K08.8- K14.9, M26-M27.9 in International Statistical Classification of Diseases and Related Health Problems, 10th revision (ICD-10)) is subcategorised into dental caries (either deciduous or permanent teeth) (ICD-10 code: K02.0-K02.9), periodontal diseases (ICD-10 code: K05-K06.9), edentulism and severe tooth loss (ICD-10 code: K08.0-K08.499), and other oral disorders (ICD-10 code: K00-K01.1, K03-K04.99, K07-K08, K08.8-K14.9, M26-M27.9) [13]. Oral disorders are also identified with the B.12.6 code in the GBD database. Detailed information on the analytical methods for estimating the burden of oral disorders can be found elsewhere [3, 13].

We defined two indices related to the quality of care, as follows:

1. $$\textrm{YLD}\ \textrm{to}\ \textrm{incidence}\ \textrm{ratio}=\frac{\#\textrm{YLDs}\ }{\#\textrm{Incidence}}$$
2. $$\textrm{Prevalence}\ \textrm{to}\ \textrm{incidence}\ \textrm{ratio}=\frac{\#\textrm{Prevalence}\ }{\#\textrm{Incidence}}$$

These two indices can follow a particular trend individually. Lower values of YLDs to incidence ratio could mean the lower burden of disease and/or lower occurrence of the cases, which is further expounded as better care and/or better prevention. Prevalence to incidence ratio shows the effectiveness of prevention programs (if any) in which lower values imply a better oral health status [14]. Among these indices, the trend of each one can easily be determined. For instance, higher values of prevalence to incidence ratio could mean better care and/or better prevention among patients. Higher prevalence to incidence ratio suggests higher prevalence in a fixed incidence rate, which is indicative of more accurate diagnosis, prolonged life span, and better care quality. DALYs to prevalence ratio is high when a high burden of the disease (due to either mortality or morbidity) is present in the country. Higher values of the DALYs to prevalence ratio suggest a greater burden and disability in a specific prevalence rate.

To make generalizable inferences from these individual indices, we summarized them using principal component analysis (PCA). This method is a multivariate analytical approach that extracts linear combinations of variables as either orthogonal or uncorrelated components [15]. The first-ranked component of both of these indices in the PCA analysis was considered as the quality-of-care Index (QCI). Component scores were calculated on a scale from 0 to 100 in which higher numbers indicate a better status [16].

Other than the overall QCI for oral disorders, it was separately calculated for caries of deciduous teeth, caries of permanent teeth, periodontal diseases, and edentulism. While assessing the distribution of QCIs, we categorized the regions based on two approaches: Socio-demographic Index (SDI) classification [17]. This classification can represent the development status of a region based on various factors (i.e., economic average incomes per capita, average educational attainment and fertility rates). To assess gender inequality in each country, we used gender disparity ratio (GDR), which is the male to female ratio of QCIs. Concerning the GDR values, five quintiles were defined: 0 to 0.5, 0.5 to 0.95, 0.95 to 1.05, 1.05 to 1.5, and more than 1.5. We considered the 0.95 to 1.05 quintiles as the optimum GDR. Six-sigma approach was applied to find the outlier countries (with very high or very low QCIs). The outlier countries could represent two situations: either a weak performance in a specific condition such as outbreaks, or a significantly higher or lower prevalence of a condition. In doing so, we calculated the mean and standard deviation of QCI and identified the values out of the “(*μ* − 3*σ*, *μ* + 3*σ*)” range –as the outliers. This methodology can be found in further details elsewhere [18]. From here onwards, all the QCIs mentioned in this paper, that are not accompanied by a specified age category, are age-standardised. For age disparity patterns, the QCI for each age group was calculated separately on global and SDI scales. We considered ages under 20 years as “childhood and adolescence”, 20–65 as “adulthood”, and above 65 as “the elderly”.

### Validation

We applied a mixed-effect regression model on QCI as a dependent variable and inpatient and outpatient health care utilisation and prevalence (of oral disorders) as independent variables. In this regard, countries were considered as a random effect and a Pearson’s correlation coefficient was calculated between the predicted QCI and Healthcare Access and Quality Index (HAQI) [19], an index to evaluate the accessibility of care [14, 20].

### Statistical analysis

We used R 3.6.0 (R Core Team, 2019) to perform the analyses. Detailed information on the steps of our mathematical methods and the statistical protocol are provided elsewhere [21].

## Results

### Burden of oral disorders

Globally, oral disorders caused more than 18.3 million YLDs (95% Uncertainty Interval (UI): 11.0–28.3) (compared to 10.9 million YLDs (6.6–16.8) in 1990) in 2017. YLDs rates continuously increased through the years and reached an average rate of 228.8 (137.5–353.7) in 2017 (compared to 238.3 [145.5–366.0] in 1990). Different subcategories of oral disorders (except for “other oral disorders” subcategory) caused the following YLDs in all ages in 2017 worldwide: 138,916 for caries of deciduous teeth, 1,618,887 for caries of permanent teeth, 5,185,589 for periodontal diseases, 7,345,908 for edentulism and severe tooth loss. Oral disorders were responsible for 0.73% (0.46–1.08%) of global YLDs in 2017 (compared to 0.42% (0.27–0.63%) of total YLDs in 1990). YLDs rate for women was higher than men in 2017 .1 [119.1–315.7]). Based on World Bank regions, from 1990 to 2017, the YLDs rate of all oral disorders slightly decreased in Europe and Central Asia (from 293 to 288 per 100,000), North America (from 236 to 214 per 100,000), and Latin America and Caribbean (from 336 to 335 per 100,000). During the same period, the prevalence of oral disorders has decreased in all World Bank regions.

The global age-standardized YLDs rate of caries of deciduous teeth decreased from 2.3 (1.0–4.4) in 1990 to 2.0 (0.9–4.1) in 2017. The burden of permanent teeth caries also decreased from 23.4 (10.2–44.6) in 1990 to 20.7 (8.9–39.2) in 2017. Worldwide, YLDs of periodontal diseases increased from 59.9 (23.6–123.3) in 1990 to 63.5 (25.0–130.3) in 2017. For edentulism and severe tooth loss, it also increased from 102.0 (68.2–143.5) in 1990 to 91.7 (61.3–129.9) in 2017. YLDs of other oral disorders also increased, from 50.8 (31.7–74.5) in 1990 to 50.9 (31.8–74.7) in 2017 (Table 1).

**Table 1:** Age-standardised estimates of burden and QCI of oral disorders globally and by SDI quintiles

	DALYs rate in 2017 (per 100,000)	DALYs change 1990 to 2017 (%)	QCI in 2017 (%)	QCI change 1990 to 2017 (%)
**Global**				
**All Oral Disorders**	228.8 (137.5, 353.7)	−4.0 (− 5.7, − 2.4)	74.6	4.1
**Caries of Deciduous Teeth**	2.0 (0.9, 4.1)	−9.0 (− 11.0, − 7.3)	81.89	2.69
**Caries of Permanent Teeth**	20.7 (8.9, 39.2)	−11.9 (− 13.4, − 10.3)	77.2	7.7
**Periodontal Disease**	63.5 (25.0, 130.3)	6.0 (5.2, 6.8)	71.1	−2.2
**Edentulism**	91.7 (61.3, 129.9)	−10.1 (− 10.9, −9.3)	61.2	−5.0
**Other Oral Disorders**	50.9 (31.8, 74.7)	0.2 (− 0.1, 0.6)	*NA*	*NA*
**SDI Quintiles**				
**High SDI Quintile**				
**All Oral Disorders**	219.4 (136.0, 332.8)	−16.2 (−17.7, − 14.9)	78.03	13.77
**Caries of Deciduous Teeth**	2.2 (1.0, 4.5)	−7.7 (− 12.2, − 3.7)	84.15	3.43
**Caries of Permanent Teeth**	27.6 (12.0, 53.6)	−10.9 (− 12.2, −9.5)	69.46	12.15
**Periodontal Disease**	42.1 (16.6, 86.7)	−15.3 (− 17.3, − 13.2)	73.26	−1.02
**Edentulism**	96.7 (64.3, 138.1)	−24.3 (− 25.5, − 23.0)	75.70	−0.38
**Other Oral Disorders**	50.8 (31.7, 75.3)	−0.1 (− 0.6, 0.5)	*NA*	*NA*
**High-Middle SDI Quintile**				
**All Oral Disorders**	238.4 (146.8, 364.1)	−4.7 (− 6.2, − 3.3)	80.24	5.81
**Caries of Deciduous Teeth**	2.5 (1.1, 4.9)	−4.1 (−7.5, −1.6)	80.88	2.65
**Caries of Permanent Teeth**	20.9 (9.1, 39.9)	−12.6 (− 13.8, − 11.0)	83.24	8.27
**Periodontal Disease**	56.9 (22.4, 116.5)	5.2 (3.8, 6.5)	78.92	−4.04
**Edentulism**	107.1 (71.6, 151.6)	−9.7 (−10.4, −9.1)	78.86	1.84
**Other Oral Disorders**	51.0 (31.8, 75.3)	0.0 (−0.6, 0.6)	*NA*	*NA*
**Middle SDI Quintile**				
**All Oral Disorders**	237.2 (141.3, 369.0)	4.4 (3.8, 5.1)	74.96	1.47
**Caries of Deciduous Teeth**	2.4 (1.0, 4.7)	−2.1 (−5.0, 0.4)	79.93	1.97
**Caries of Permanent Teeth**	20.3 (8.8, 38.0)	−5.3 (−6.7, −3.8)	80.06	5.09
**Periodontal Disease**	69.6 (27.5, 142.0)	5.7 (4.8, 6.6)	70.00	−0.85
**Edentulism**	93.8 (62.5, 133.0)	8.5 (7.7, 9.2)	81.45	−0.48
**Other Oral Disorders**	51.1 (31.9, 75.5)	0.3 (−0.2, 0.8)	*NA*	*NA*
**Low-Middle SDI Quintile**				
**All Oral Disorders**	228.3 (134.7, 358.0)	6.0 (4.9, 7.2)	71.52	−0.37
**Caries of Deciduous Teeth**	1.7 (0.7, 3.5)	−11.5 (−13.9, −8.9)	84.90	1.88
**Caries of Permanent Teeth**	16.7 (7.3, 31.6)	−10.3 (− 11.5, − 8.9)	80.27	6.90
**Periodontal Disease**	76.7 (30.3, 155.5)	7.0 (5.6, 8.5)	61.76	−1.92
**Edentulism**	82.4 (54.8, 115.9)	13.4 (12.4, 14.4)	81.11	−1.53
**Other Oral Disorders**	50.7 (31.7, 74.4)	0.6 (−0.2, 1.4)	*NA*	*NA*
**Low-Middle SDI Quintile**				
**All Oral Disorders**	201.5 (115.0, 324.1)	5.7 (4.6, 6.6)	70.51	0.53
**Caries of Deciduous Teeth**	1.8 (0.8, 3.6)	−4.9 (−8.0, −1.9)	83.89	1.44
**Caries of Permanent Teeth**	19.3 (8.4, 36.1)	−5.7 (−7.2, − 4.5)	69.55	5.78
**Periodontal Disease**	75.6 (29.8, 154.7)	10.1 (8.0, 11.6)	57.83	−3.48
**Edentulism**	54.2 (35.9, 76.7)	9.6 (7.9, 11.1)	83.56	−1.34
**Other Oral Disorders**	50.6 (31.7, 74.2)	0.8 (−0.1, 1.7)	*NA*	*NA*

*NA,* not applicable

### Quality of care index and gender inequity

**Fig. 1:**
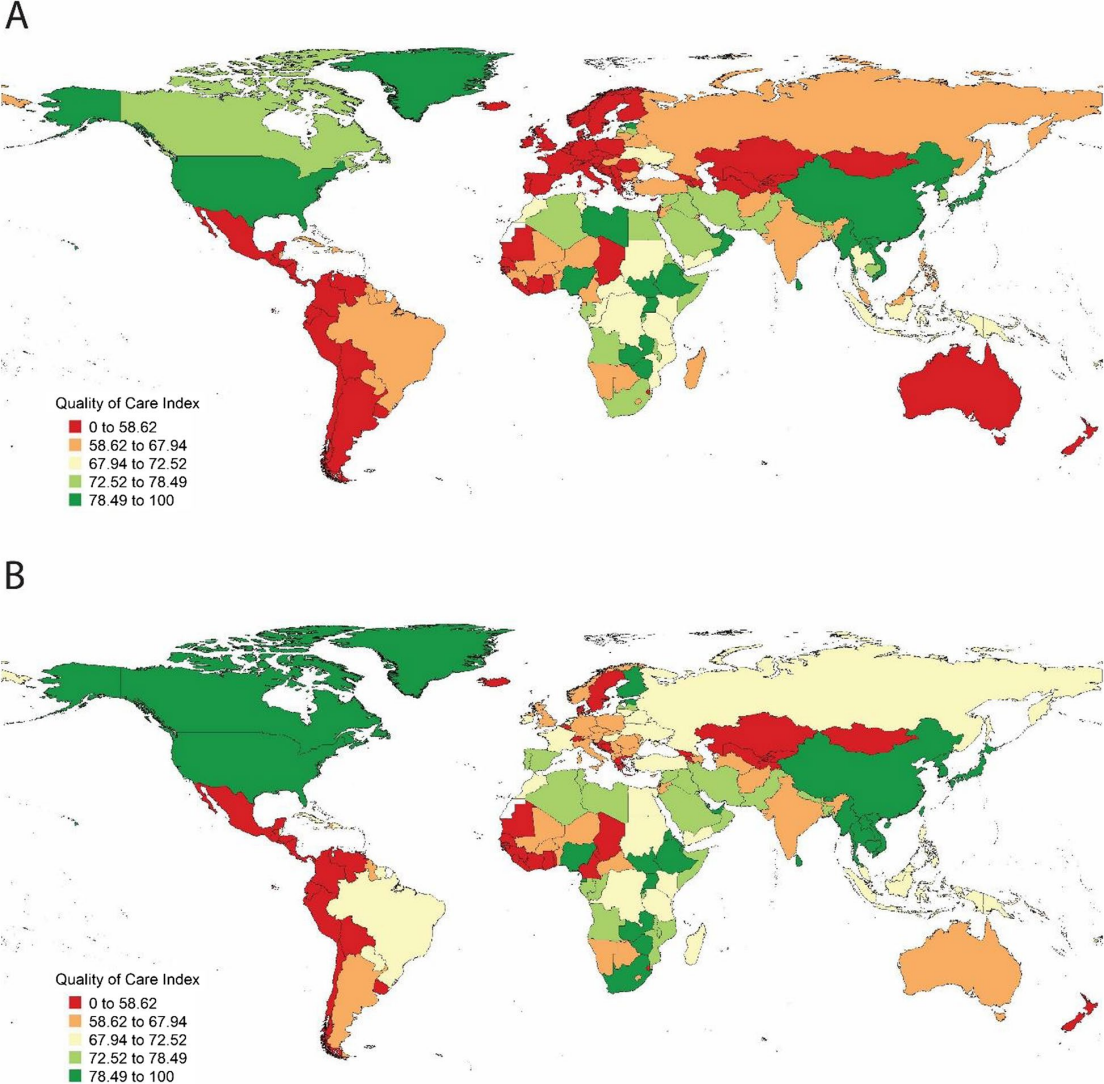
Geographical distribution of QCIs^*^ (%) for oral disorders. **A** Global distribution of age-standardised QCI in men and women in 1990, **B** Global distribution of age-standardised QCI in men and women in 2017. QCI: Quality of care index

**Fig. 2:**
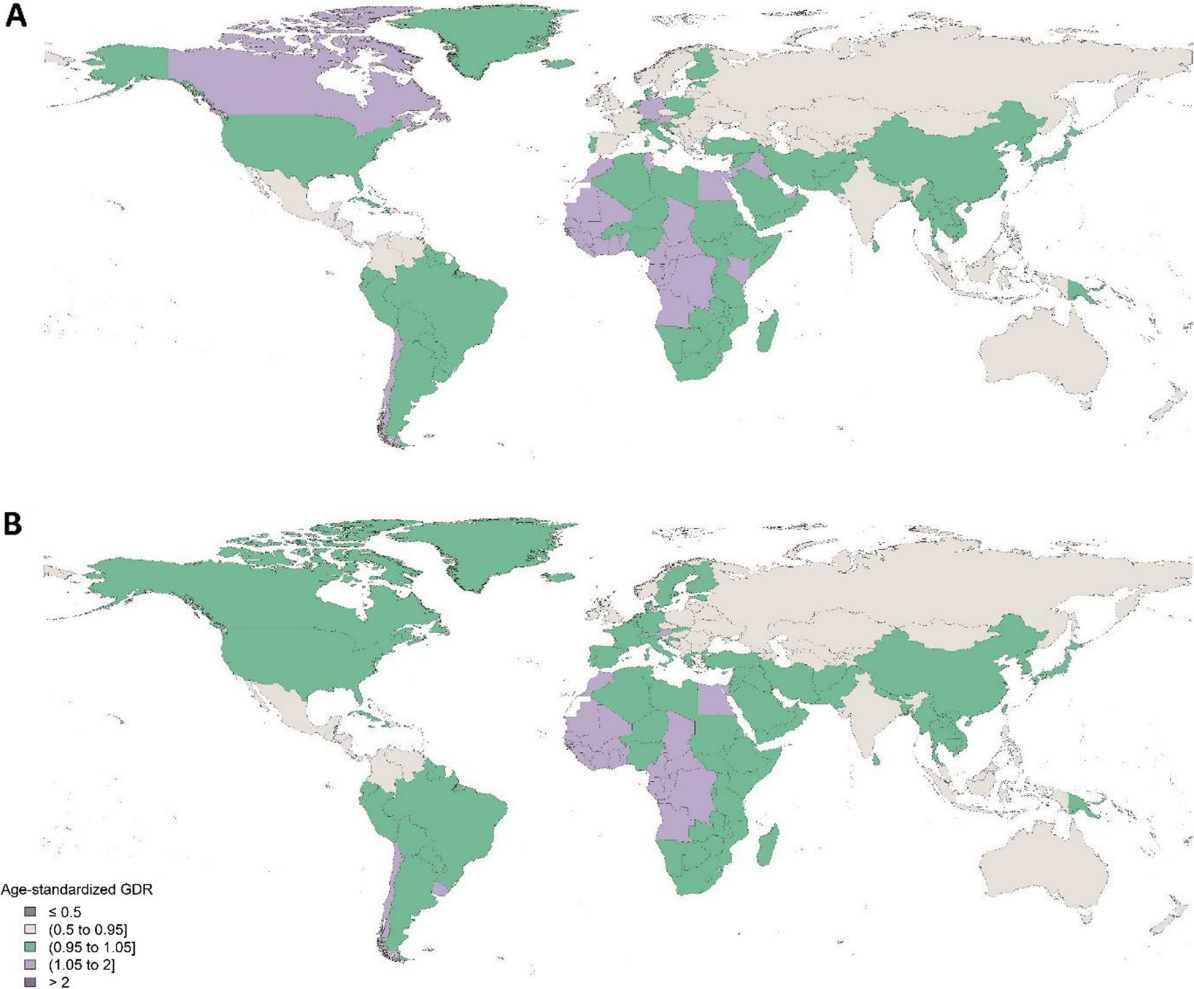
Geographical distribution of GDR for oral disorders. **A** Age-standardised gender disparity ratio in men and women in 1990, **B** Age-standardised gender disparity ratio in men and women in 2017. GDR: Gender disparity ratio

Global QCI for oral disorders gradually increased from 1990 to 2017 from 70.5 to 74.6 (Fig. 1). Quality of care for women was lower than men (68.2 compared with 72.2) in 1990 worldwide. It then increased for both men and women globally throughout this period. However, women still experienced lower care quality in 2017 (72.5 compared with 76.0) (Fig. 2). The gender disparity ratio was 1.05 in both 1990 and 2017.

**Fig. 3:**
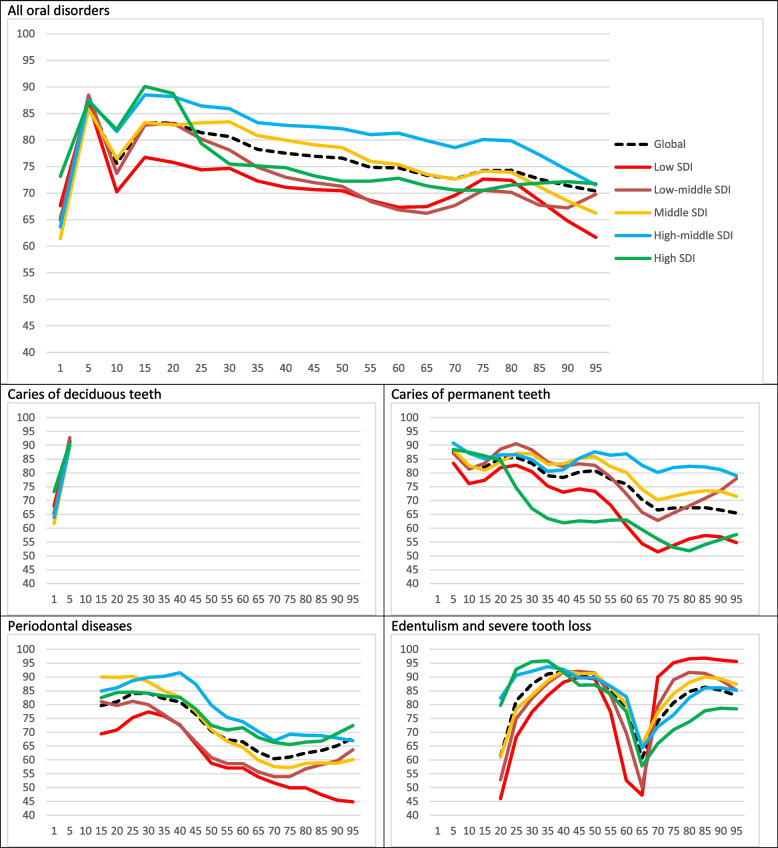
Disparity patterns of oral disorders in various global and socio-demographic index (SDI) quintiles in 2017. Vertical axis represents the QCI scores (from 0 to 100) in both sexed combined, while the horizontal axis shows the age number. Distinct colours distinguish trends in various SDI quintiles and the global trend

**Fig. 4:**
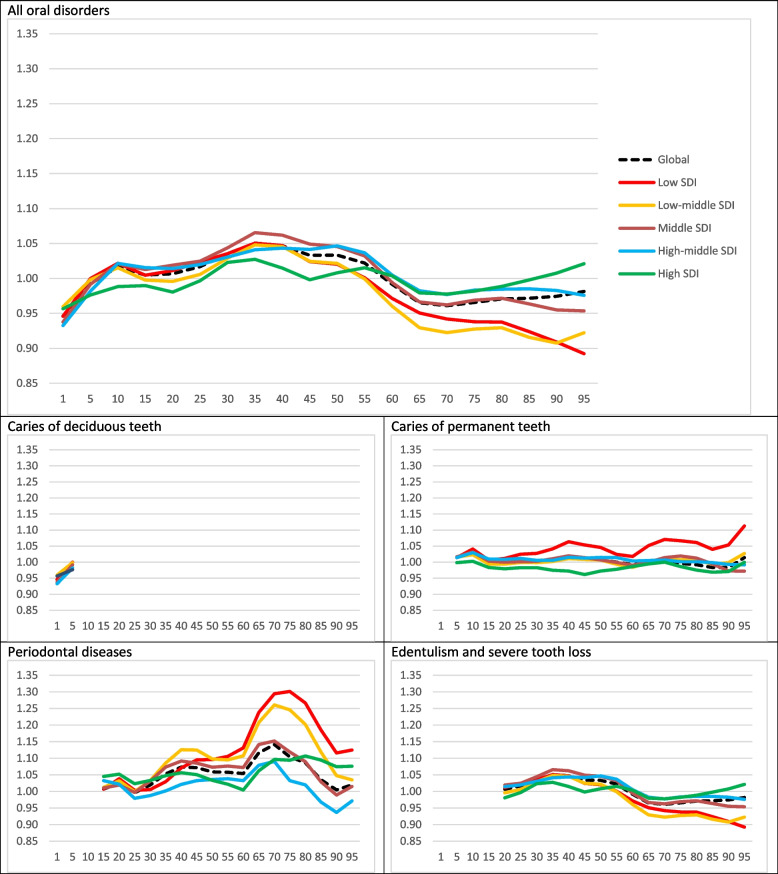
Disparity patterns of oral disorders in various global and socio-demographic index (SDI) quintiles in 2017. Vertical axis represents the gender disparity ratio (GDR) in both sexes combined, while the horizontal axis shows the age number. Distinct colours distinguish trends in various SDI quintiles and the global trend

Among all the age groups, the QCI of oral disorders in 5–9-years-old was the highest in both 1990 and 2017 (86.0 and 87.2, respectively). Overall, the adulthood age group showed a higher quality of care compared with the elderly age group. The QCI in 1–4-years-old children, as the age group with the least QCI score for oral disorders, increased from 62.3 to 65.1 in the years 1990 and 2017, respectively. It followed the same pattern in 5–9-years old children and increased from 86.0 in 1990 to 87.2 in 2017. Globally, the QCI of caries of permanent teeth also increased from 69.5 in 1990 to 77.2 in 2107. Turning to periodontal diseases and total tooth loss, the QCI for periodontal diseases decreased from 73.3 to 71.1 throughout these years, whereas slightly dropping for edentulism, from 66.2 to 61.2 (Table 1). Concerning gender disparity, all the age groups had an optimum GDR score worldwide. On a global scale, the highest GDR was observed in the 35–40 age group (adulthood) at a figure of 1.05. The GDR score bottomed at 0.89 in the low-SDI quintile (countries) in the elderly age group (95 years old) whereas peaking at 1.07 in the middle-SDI quintile in 35–years old. Figures 3 and 4 show the QCI and GDR trend among distinct age groups in all the SDI quintiles and worldwide.

### Quality of care index among the countries

**Fig. 5:**
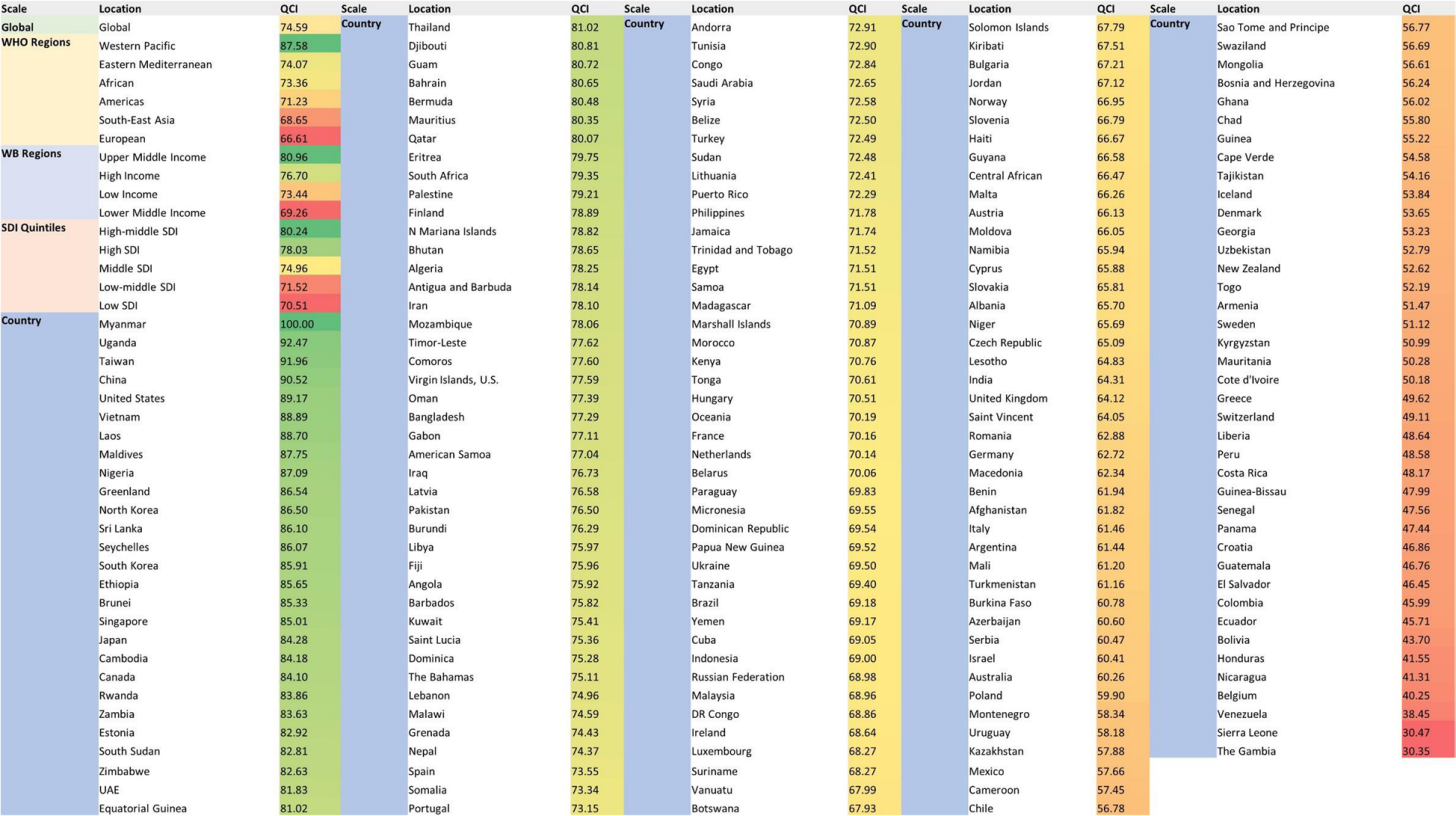
Global regions and countries listed in a descending order based on their QCIs for oral disorders

Between 1990 and 2017, the age-standardised QCI for all oral disorders increased in all the SDI regions. According to the SDI classification, while the highest QCI for all oral disorders in 2017 belonged to high-middle SDI countries (=80.24), the lowest YLDs rate was seen in the low SDI quintile (Fig. 5). The following five countries enjoyed the highest increases in QCI from 1990 to 2017: France (34.8), Belgium (31.4), Spain (30.8), Israel (29.3), and Germany (29.3). These five countries, on the other hand, had the highest decreases in QCI in the same period: Nigeria (− 11.4), Guinea-Bissau (− 10), Western Sub-Saharan Africa (− 9.6), Libya (− 9.4), and Guinea (− 7.7).

Maynmar (=100), Uganda (=92.5), Taiwan (=92.0), China (=92.5), and the United States (=89.2) were the five countries with the highest age-standardised QCI scores among the others in 2017. On the other hand, Nicaragua (=41.3), Belgium (=40.2), Venezuela (=38.4), Sierra Leone (=30.5), and the Gambia (=30.3) were the five countries with the least age-standardised QCI values in 2017. While the QCI for all oral disorders has increased in the European and South African countries during these 28 years, Belgium’s index was three sigmas lower than the average from 1990 to 2005. Figure 5 shows the global, regional, and country by country QCI in 2017.

## Discussion

This study introduces an original multivariate index (the QCI) that strives to show the quality of care for oral disorders worldwide from 1990 to 2017. To our knowledge, no study has yet focused on the quality of care in the oral health context on a global scale. Therefore, we tried to applicate the most comprehensive measures to capture different aspects of oral health care as it can be salient for policymakers that aim to enhance the oral health status and its quality of care among populations worldwide.

We defined oral disorders as all the pathologic conditions occurring in the oral cavity except for the lip and oral cavity cancers. None of these conditions —including teeth caries, periodontal diseases, edentulism, and other disorders— directly leads to mortality. Additionally, oral disorders are long-lasting conditions, and their management has repeatedly proven to be a challenge for health care systems around the world [1, 21, 22].

Several wide-scale policies have been proposed to mitigate the burden of oral disorders on the healthcare systems, including adding dental care to universal health coverage with the help of minimal intervention dentistry, pursuing preventive approaches to intercept oral disorders at the initiation phase, controlling shared risk factors (with other systemic diseases), and managing the social and commercial determinants of oral disorders [1, 21, 23]. As multiple risk factors like diet, hygiene, smoking and alcohol consumption, stress, and trauma, are suggested as common risk factors mainly affecting the oral health status [1, 24, 25], the proposed policies to improve oral health should also focus on these risk factors [25–29].

Policymakers in the oral health sector are encouraged to address not only the direct risks but also broader and more fundamental issues in the hierarchy of risk factors for oral health disorders. Providing high-quality oral health care can notably enhance the quality of life (decreased YLDs) and reduce the prevalence of these conditions among the public [30]. All these approaches can eventually result in more patients getting quality care for their oral disorders. Our index, the QCI, includes public-health measures to estimate a proxy for the quality of care in various global countries and regions.

We found that the overall trend of QCI was upwards for oral disorders globally, a sign of moving towards a better quality of care in all regions (classified by SDI). For all oral disorders, the lowest quality of care was seen in a diverse set of countries (Central Africa, Central and Latin America, and Western Europe). This is in line with the findings of the GBD study 2017, which found a similar distribution for normative need and YLDs of oral disorders in various regions [21]. Cote d’Ivoire, a low-income country, was among the 10 countries with the least QCIs for both periodontal diseases and edentulism, while not being one of the 20 countries with the least QCIs for all oral disorders. This finding can be explained by the fact that low-income countries had the least burden of untreated caries in 2017 —as the traditional diet is low in carbohydrates [31, 32]— with a cost-efficient choice of symptom relief in patients (tooth extraction) [33]. Economic conditions likely restricted their access to effective treatments for periodontal diseases or complete dental loss (via periodontal rehabilitation or prosthetic options) [34–36].

For all oral disorders, among the European countries, Belgium was the only one with a QCI value three sigmas lower than the average from 1990 to 2005. It had the fourth-lowest QCI in 2017 for all oral disorders as well. Upon more in-depth inspection, we found that nearly 43% of YLDs rate was attributed to edentulism. Because patients in Belgium were more likely to wear a removable denture (versus more sophisticated treatment plans tending to rehabilitate the chewing functions more effectively), the lower QCI for edentulism can be rationalised [37]. Higher YLDs rate of total tooth loss is associated with tooth death spiral, a phenomenon most likely to happen in more affluent countries with usually better access to dental services and oral healthcare [38–40]. Although high-income countries have been trying to adopt more preventive approaches for dental treatments, the YLDs rates of deciduous teeth caries were lower in regions with lower SDI status. Genetic and nutritional (low-carbohydrate diet) factors both contribute to lower burden and accordingly lower QCIs of caries in deciduous teeth in “low-status” countries [31, 41, 42].

In 2017, while having the least YLDs rate for tooth caries and periodontal diseases, middle-high and high SDI countries generally showed higher QCI values for periodontal diseases than those with low or low-middle status. This fact can be expounded by knowing that these countries have already reduced their sugar consumption [43] and naturally have better access to more complex periodontal treatments [44].

Looking at the QCI’s global age patterns, interestingly, two peaks occurred in the quality of care, both happening in the children and adolescents age group. This reflects the success of preventive approaches in below-20-year populations to reduce the prevalence and burden of oral disorders worldwide. Notably, low- and low-middle SDI countries showed lower peaks than their counterparts. Globally, the quality of care for all oral disorders fluctuated on a downward trend with advancing age. This supports the growing concerns to provide higher quality dental care for the adulthood and elderly demographics and meet their needs around the world [45]. Caries of permanent teeth showed quite a similar QCI pattern among the age groups, implying a need to heed the permanent teeth and their perpetuating issues in older individuals [46]. However, the QCI plummeted at 65–70-year-olds for periodontal diseases and edentulism, then rising toward higher figures. The plunging and recovery were sharper for edentulism. These results can be explained by the fact that, most likely, the number of remaining teeth had already decreased to its lowest before the initiation of “the elderly” age group. This essentially means a natural alleviation for periodontal problems (as the teeth with unfavourable prognosis had already been lost). After this stage, the elderly patients will probably seek prosthetic solutions and rehabilitative treatments more actively, hence the sharp increase in the QCI of edentulism in those older than 70.

Regarding the gender disparities, the GDR was optimal both in 1990 and 2017 for all oral disorders worldwide. This can be positive finding as there was no significant disparities between men and women regarding the QCI of oral disorders throughout these years.

Our results can help develop oral healthcare provision strategies (either local or national) and provide evidence to guide health policy-making procedures in the future. As such, the importance of pursuing preventive and minimal intervention dentistry (and avoiding the death spiral cycle) in all regions of the world is reiterated. Prioritising the quality of care for edentulism in more affluent areas while enhancing access to primary healthcare in more deprived areas (to halt tooth caries) is also a key takeaway from our results. More research with a specific focus on the quality of care for each oral disorders (i.e., dental caries, periodontal diseases, edentulism) is highly recommended.

### Limitations

It should be reiterated that QCI values with distinct causes are not comparable, and one should only compare the QCI values within the confines of the same cause (in different times and locations). We tried to cover various aspects of oral healthcare in the context of the GBD database. The major factors to consider were: DALY, mortality, prevalence, and incidence of the disease. Nevertheless, considering our data’s restrictions, other factors influencing the quality of care were out of reach to evaluate (such as patient satisfaction, staff responsiveness, treatment reliability and validity assessment, etc.). Furthermore, the accessibility of healthcare was not considered directly in our index. Our results are better to be interpreted cautiously as they are relative estimations and do not report absolute figures.

## Conclusion

The quality of care for all oral disorders showed an increasing trend on a global scale from 1990 to 2017. Nevertheless, the QCI distribution was not homogenous among various regions. To tackle this issue, better attention to total tooth loss in high-income countries and prioritising primary healthcare provision in low-income countries are recommended.

### Supplementary Information

**Additional file 1.**

## Data Availability

Preliminary, we used GBD 2017 dataset, which is publicly available, to generate the data for our statistical analysis (available at: https://gbd2017.healthdata.org/gbd-search). The generated datasets are available at Supplementary Materials.

## Abbreviations

GBD: Global Burden of Disease
YLLs: years of life lost
YLDs: years lived with disability
DALYs: disability-adjusted life years
PCA: principal component analysis
SDI: Socio-demographic Index
GDR: gender disparity ratio
QCI: quality-of-care Index
HAQI: Healthcare Access and Quality Index
