# Correction: Global, regional, and national burden and quality of care index (QCI) of oral disorders: a systematic analysis of the global burden of disease study 1990–2017

**DOI:** 10.1186/s12903-024-04236-3

**Published:** 2024-05-02

**Authors:** Shervan Shoaee, Erfan Ghasemi, Ahmad Sofi‑Mahmudi, Erfan Shamsoddin, Marcos Roberto Tovani‑Palone, Shahin Roshani, Mohammad‑Hossein Heydari, Moein Yoosefi, Masoud Masinaei, Sina Azadnajafabad, Esmaeil Mohammadi, Negar Rezaei, Bagher Larijani, Hossein Fakhrzadeh, Farshad Farzadfar

**Affiliations:** 1 Non‑Communicable Diseases Research Center, Endocrinology and Metabolism Population Sciences Institute, Tehran University of Medical Sciences, Tehran, Iran; 2 Elderly Health Research Center, Endocrinology and Metabolism Population Sciences Institute, Tehran University of Medical Sciences, Tehran, Iran; 3 Endocrinology and Metabolism Research Center, Endocrinology and Metabolism Clinical Sciences Institute, Tehran University of Medical Sciences, Tehran, Iran; 4 Cochrane Iran Associate Centre, National Institute for Medical Research Development (NIMAD), Tehran, Iran; 5 Ribeirão Preto Medical School, University of São Paulo, São Paulo, 14049‑900 Brazil; 6 The Netherlands Cancer Institute (NKI), Amsterdam, Netherlands; 7 School of Dentistry, Shahid Beheshti University of Medical Sciences, Tehran, Iran; 8 Department of Epidemiology and Biostatistics, Tehran University of Medical Sciences, Tehran, Iran

**Correction** **:** **BMC Oral Health 24, 116 (2024)**

**https://doi.org/10.1186/s12903-023-03808-z**

In this article [1], the author name Sina Azadnajafabad was incorrectly written as Sina Azadnaejafabadi and Hossein Fakhrzadeh should be denoted as co-corresponding author.
